# On a New Source of Kino

**Published:** 1853-04

**Authors:** Robert Christison

**Affiliations:** Professor of Materia Medica in the University of Edinburgh


### MATERIA MEDICA AND THERAPEUTICS.

*On a new source of Kino.* By ROBERT CHRISTISON, M. D., V. P. R. S. E., Professor of Materia Medica in the University of Edinburgh.—In a letter of the 20th of last July, from a merchant of Moulmein, Mr. R. S. Begbie, son of Dr. Begbie of this city, I was informed that a species of kino—which seemed to him to present the physical and chemical properties of the commercial variety of that drug in the English home market, and which had been ascertained by a medical friend at Moulmein to possess also its medical virtues—might be largely obtained from a tree abounding in the adjacent provinces. Mr. Begbie added, that he believed "a small quantity had been sent some years ago to England; but as an article of export generally it has not yet been shipped." This notice was accompanied by a small specimen, which is now produced, and which is large enough to allow of its principal properties being accurately ascertained.

As the inquiries I have made lead me to suppose that the article in question is of a very fine kind, and that the fact of its production near Moulmein, and probably over a considerable part of the neighboring province of Pegue, is not hitherto known in Europe, I beg to present to the Pharmaceutical Society the following description of it, and the reasons which induce me to think that it is obtained from the identical tree which yields in Malabar the present commercial kino of European trade.

The small portion sent by Mr. Begbie consists partly of little angular fragments; but there are several larger masses which are portions of cylinders, about half an inch in diameter, apparently moulded by collecting the juice in reeds. These have externally a greyish, striated surface, most unlike that of the broken fragments of commercial kino. They are easily frangible; and the broken pieces have exactly the appearance of ordinary kino, except that they are even blacker, and more glassy by reflected light; and by transmitted light, though opaque when of very moderate thickness, they are of a splendid cherry-red color in very thin fragments. They are easily reduced to fine powder, which has a dark, dirty, lake tint. Their taste is very slightly bitter and intensely astringent.

Cold water acts more quickly on this kino than on the kino of commerce, gradually dissolving a very large proportion of it, and forming a deep, cherry-red, astringent solution; and there is left a small proportion of greyish flocculent matter, which is slowly soluble in a great measure in boiling water, and which appears to be analogous to the insoluble variety of gum called bassorin. Boiling water dissolves this kino almost entirely, and the solution, when cold, continues nearly transparent for at least an hour; but afterwards it becomes slightly turbid, and a scanty, flocculent precipitate slowly subsides. Both the hot and cold solutions yield, when much diluted, a deep, olive-green precipitate with the tincture of sesquichloride of iron; and when the solution is concentrated, a dirty grey precipitate is formed so abundantly that the whole fluid becomes a thick pulpy mass. A boiling solution in twenty-five parts of water forms with the iron test a pulp too thick to flow, which is one of the characters assigned in the Edinburgh Pharmacopœia to true officinal kino. But I find, further, that a solution in even seventy-five parts of cold water, has a beautiful, intense cherry-red color, and forms with sesquichloride of iron, in the course of an hour, a pulp so thick as to flow only sluggishly.

On comparing these characters with a fine specimen of kino of home trade, and also with a specimen collected in the neighborhood of Goomsoor, in Mysore, by Dr. Cleghorn, of the Madras Medical Service, when he was Surgeon of the surveying corps of that country, I find that the last two are identical, with the single exception, that Dr. Cleghorn's specimen is somewhat redder when seen in bulk; and that the Moulmein kino is blacker, more vitreous in lustre, rather more easily soluble in cold water, and with rather less flaky residue; and when the cold solution is diluted to the strength of one in seventy-five, it requires rather more sesquichloride of iron to throw down all its tannin, and consequently the precipitate forms with the water a somewhat firmer pulp.

This kino dissolves, with only a trace of flaky residue, in rectified spirit, which forms an intense cherry-red tincture of very pure astringent taste. The quantity in my possession is scarcely sufficient to allow of a fuller examination of its chemical properties and composition. But its physical characters, the action of water, and the properties of the watery solution, even as I have shortly indicated them, are enough to prove that the Moulmein kino is identical in nature with the present kino of home trade, and in point of quality somewhat superior. I have no doubt, from its taste, and the action of the iron test, that an analysis will prove the presence of a larger proportion of tannin.

It does not absolutely follow, even from the exact correspondences now mentioned, that the Moulmein kino is derived from the same botanical source with the present officinal kino of Europe. The officinal sort has been accurately referred by the separate researches of Dr. Gibson, Dr. Pereira, and Dr. Royle, to the *Pterocarpus Marsupium*, of Roxburgh, a fine forest tree abounding in the hills of Mysore and other parts of the Indian Peninsular. But the *Butea frondosa* also yields a fine kino, which I have shown in my Dispensatory to be scarcely distinguishable in chemical properties from the officinal kind.

Mr. Begbie, however, has fortunately supplied me with a description of the Moulmein tree, sufficient to identify it with the true kino tree of Mysore. "It is," said he, "one of the commonest trees in the adjoining provinces, and is called by the Burmese *Padouk.* It grows to a great size and height. Immediately before the rainy season it is covered with long pendant yellow flowers, of an exceedingly sweet odor, like that of jessamine. The tree flowers three times, at intervals of perhaps a week or ten days; each blow lasting about twenty-four hours. The wood is in color like mahogany, and exceedingly heavy. It is used in India for making gun-carriages; and at present we are preparing some for the London market, in executing an order, I fancy, for the Royal Artillery. It makes most beautiful furniture. The gum exudes slightly without incision; but on a cut being made into the tree, it bleeds most freely." This description is not sufficiently botanical to enable me to determine the tree from its characters in botanical works. But on submitting Mr. Begbie's letter to Dr. Gibson, Conservator of the Forests of Bombay, who very lately visited Edinburgh, that gentlemen at once recognizes his old acquaintance of the Indian woods, the *Pterocarpus Marsupium*; which he was one of the first to discover to be the true source of kino, by observing that, when his companions on a shooting party cut their names into the bark of a tree beside which they had been resting, a red juice freely exuded, and concreted into a dark, astringent gum, like the kino of commerce.—*London Pharm. Journ.*, Feb. 1853.

